# Data generated by evaluating the seasonal variability and trend analysis of the solar energy resource in the Northeastern Brazilian region

**DOI:** 10.1016/j.dib.2019.104529

**Published:** 2019-09-17

**Authors:** Fernando Ramos Martins, Francisco José Lopes de Lima, Rodrigo Santos Costa, André Rodrigues Gonçalves, Enio Bueno Pereira

**Affiliations:** a Department of Marine Science, Federal University of São Paulo, UNIFESP – Campus Baixada Santista, Santos, 11070-100, São Paulo, Brazil; b Earth System Science Center – CCST, Brazilian Institute for Space Research - INPE, São José Dos Campos, 12227-010, São Paulo, Brazil

**Keywords:** Solar irradiation data, Seasonal variability, Cluster analysis, Trend analysis

## Abstract

The solar radiation is the primary energy resource for several human activities. Nowadays, the environmental issues and climate concerning are boosting the substitution of fossil fuel resources by renewable energy resources, including the adoption of solar energy for power generation. Although the solar power stands for 0.2% of the Brazilian electricity mix, the solar energy resource in the Northeastern Brazilian region (NEB) is higher than in countries where the solar energy market is already consolidated. Nowadays, it is crucial to deepen the comprehension of solar resource time and spatial variability in NEB to support and promote the solar energy market and save water to other purposes than power generation. The paper presents the data generated by Lima et al. (2019). The database, based on meteorological observations at 129 automated weather stations, provides reliable information on the spatial and seasonal variability of the incoming solar irradiation in NEB.

**Table:** Specifications Table

Subject area	*Renewable Energy, Sustainability and Environment*
More specific subject area	*Renewable Energy Assessment*
Type of data	*Table, maps and graphs*
How data was acquired	*Ground meteorological data acquired in Automated Weather Stations (AWS) operated by Brazilian Institute for Meteorology from 2008 till 2015 in 1-h time resolution.*
Data format	*Filtered and analyzed.*
Experimental factors	*Ground data quality was checked based on regional climate characteristics and time coherence analysis.*
Experimental features	*Geostatistical analysis based on ground observations.*
Data source location	*Department of Marine Science and Technology, Brazilian Federal University of São Paulo, Brazil.*
Data accessibility	*Repository name*: *Mendeley Data.* *Data identification number*: *ggb4xymxt2/1* *URL to data*: https://data.mendeley.com/datasets/ggb4xymxt2/1
Related research article	*The Seasonal Variability and Trends for the Surface Solar Irradiation in Northeastern Region of Brazil. Sustainable Energy Technologies and Assessments Journal, 35, 335–346, 2019.*

**Table:** 

**Value of the Data** • The available database can help to understand how solar energy can contribute in proposals for incentive policies and environmental agenda focused in saving water for other purposes than power generation (water, food, and energy security nexus) in the driest Brazilian region; • The database contains vital information for the evaluation of the seasonal and spatial complementarity between renewable energy resources in the Northeastern Brazilian region; • The database allows a reliable overview of the solar energy resource in the Northeastern Brazilian region based on local AWS measurements; • The database can be useful to support energy planning activities to increase the solar power share in the Brazilian energy mix.

## 1 Data

The database provides reliable information and knowledge to identify and investigate the spatial and seasonal complementarity of the solar energy resource in the Northeastern region of Brazil (NEB) [1]. The dataset is organized in folders as described in Table 1, and it is available for public access at the Mendeley Data repository. The link to reach the complete database is https://doi.org/10.17632/ggb4xymxt2.1 [2].

**Table 1:** List of folders and their data contents available for public access at the Mendeley data repository [2].

ID.	Folder name	Description
1.	Kriging Interpolation_Surface Solar Irradiation	The folder contains data and figures provided by Kriging interpolation method applied to the incoming solar irradiation data acquired in AWS operating in the Northeastern Brazilian Region. The worksheet contains seasonal averages for the surface global solar irradiation.
2.	Cluster Analysis	The folder contains the database used to feed the Cluster Analysis (CA) script. The CA, based on the agglomerative hierarchical Ward method, identified five areas in the Northeastern Brazilian region presenting differing solar irradiation patterns.
3.	Monthly & Annual_Avgs	The folder contains the annual and monthly spatial averages of the surface global solar irradiation in all five clustered areas in the Northeastern Brazilian region.
4.	Box Plot_Dataset	The folder contains the data files used to prepare boxplot graphs of the surface solar irradiation for all clustered areas in the Northeastern Brazilian region.
5.	Trend Analysis_dataset	The folder contains the data generated in trend evaluation of the surface solar irradiation in the Northeastern Brazilian Region.

## 2 Experimental design, materials, and methods

The experimental design is based on the incoming solar irradiation data acquired at 129 automated weather stations (AWS) operating throughout NEB territory from 2008 to 2015. The Brazilian Institute for Meteorology (INMET) operates and manages all AWS in the NEB used to generate the available database in Mendeley repository [2]. The AWS data is available for free by ordering to Brazilian Institute for Meteorology (INMET) according to the instructions presented at http://www.inmet.gov.br/portal/index.php?r=bdmep/bdmep.

**Fig. 1:**
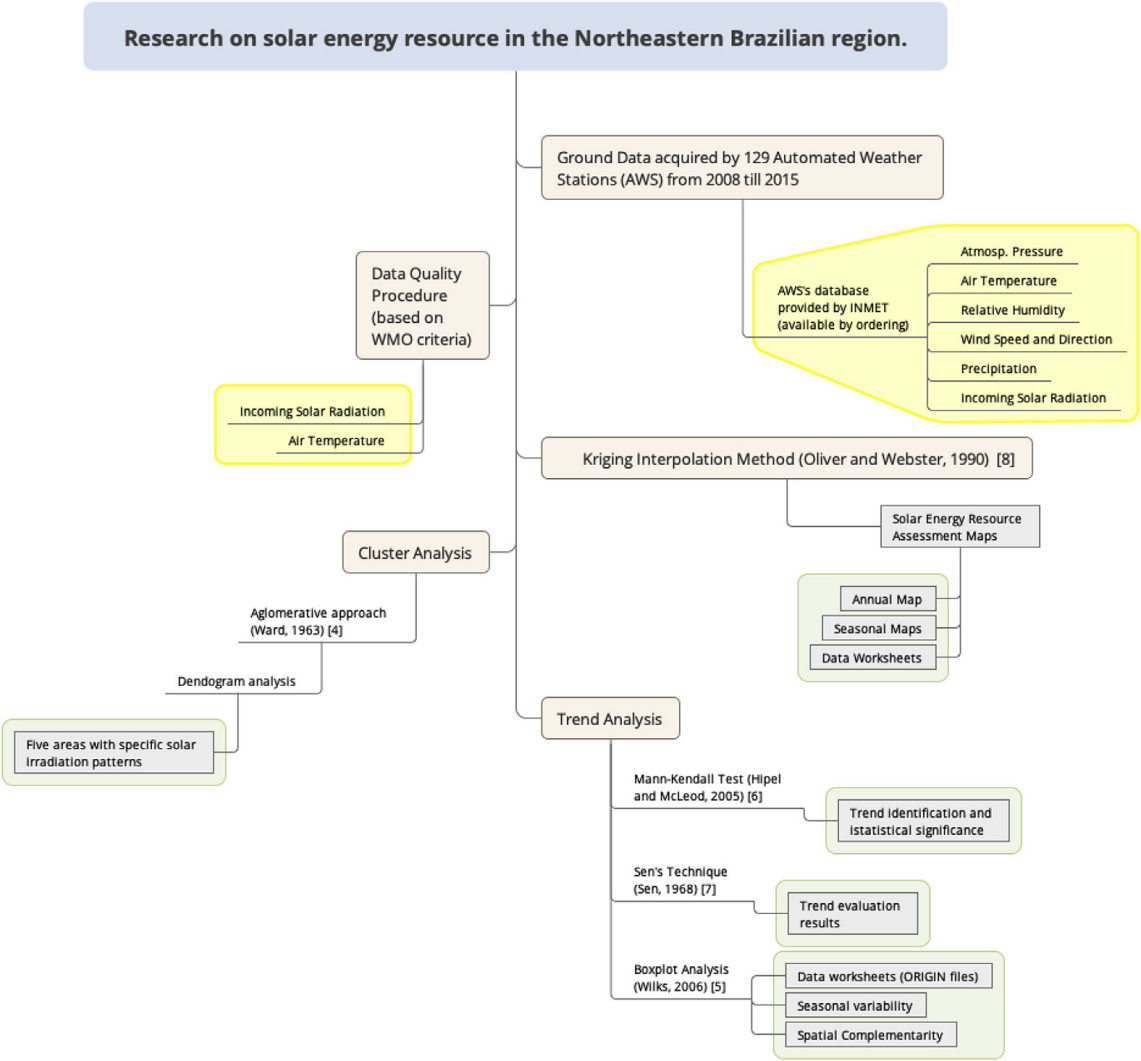
Experimental Design and methods used in evaluation of the spatial distribution pattern and seasonal variability of the incoming solar irradiation in the Northeastern Brazilian Region. The green boxes are highlighting the dataset in Mendeley repository [2]. The datasets in yellow boxes are available under demand [8].

Fig. 1 presents a diagrammatic representation of the experimental design labeling the main statistical methods used to evaluate the spatial distribution patterns and seasonal variability of the incoming global solar irradiation in the Northeastern Brazilian region. The statistical methods and tools used only reliable the incoming solar irradiation and air temperature data according to the WMO criteria [3].

**Fig. 2:**
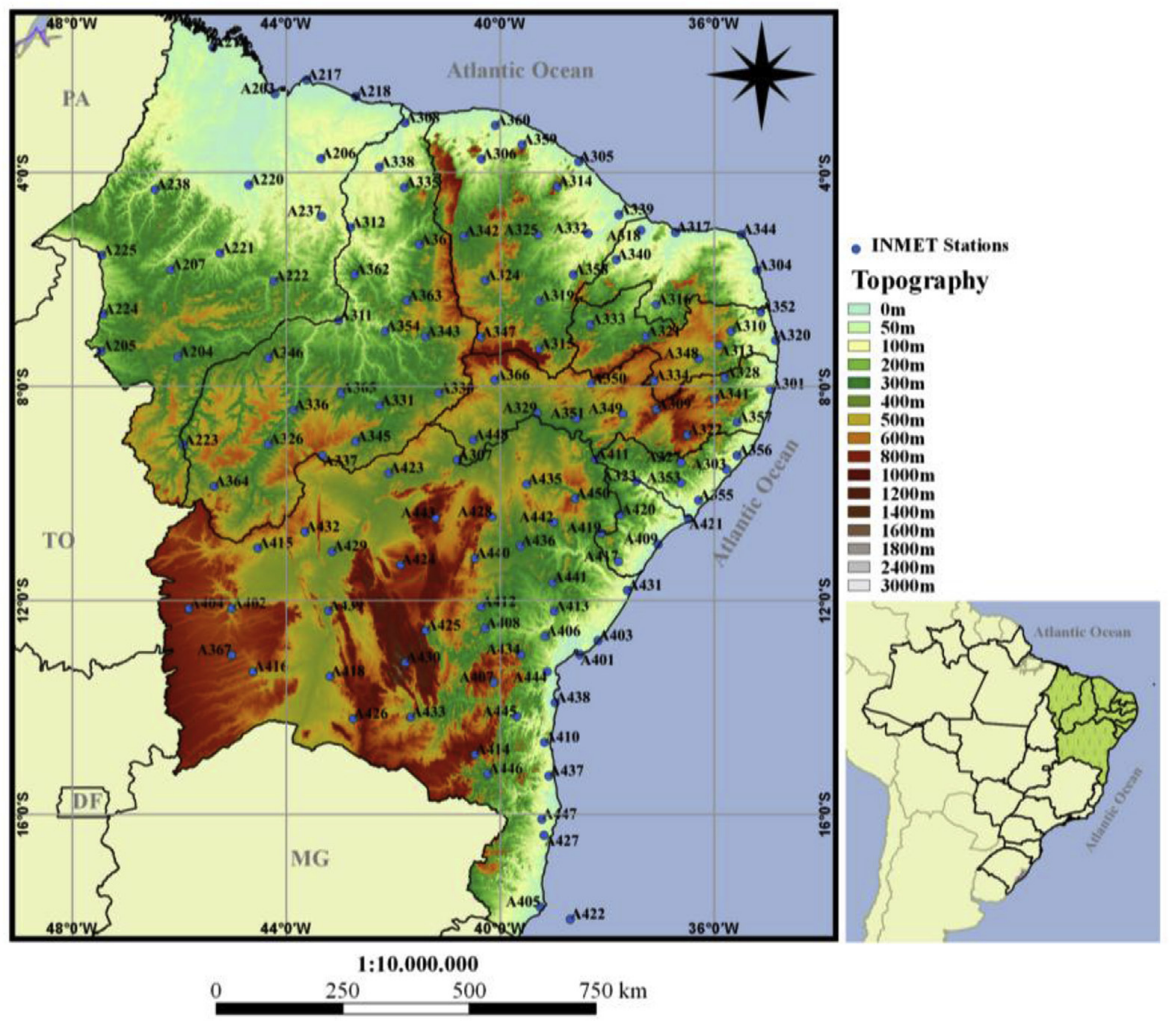
The location of 129 automated weather stations (small circles) operating in the Northeastern Brazilian region. Each AWS are named by identification code using the letter “A” followed by a number. The background colors are representing the regional topography.

Fig. 2 shows the AWS's location together with regional orography. It is important to note that the number and spatial distribution of the AWS locations provide excellent coverage of the whole territory of NEB, including areas with high altitudes.

**Fig. 3:**
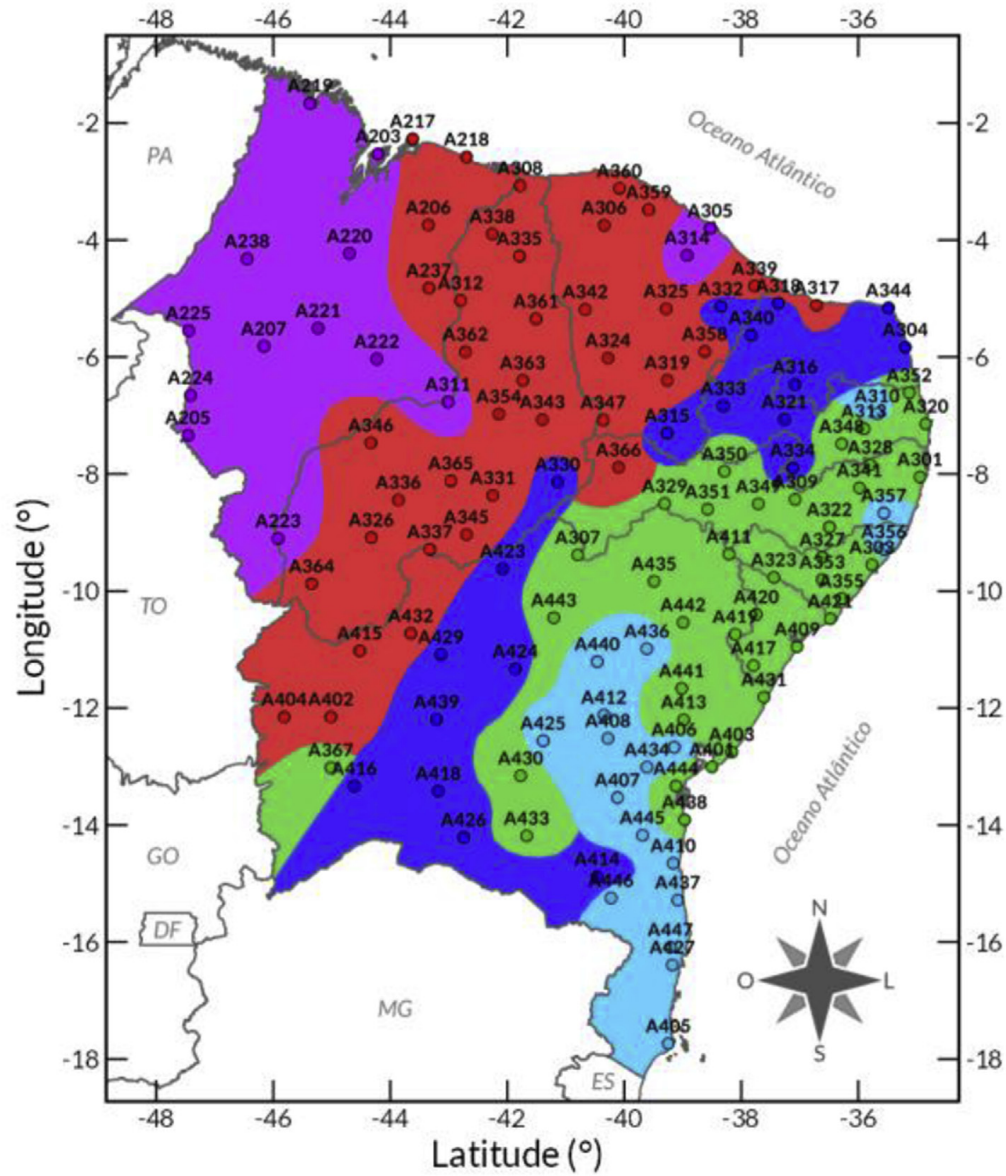
Map showing the five areas with distinctive solar irradiation patterns regarding the spatial and seasonal variability observed in NEB. The colors are outlining the geographical location of the five clusters, and dots are representing the AWS locations. Lima et al. [1] describe the patterns and seasonal variability identified for each of the five areas.

Fig. 3 presents the geographical location of areas showing similar solar irradiation regimes. The cluster analysis indicated five areas with particular global solar irradiation patterns and seasonal variability based on the agglomerative hierarchical Ward method [4]. The AWS's data located in every five areas were used to evaluate the seasonal variability [5], and trend analysis [6], [7].

**Fig. 4:**
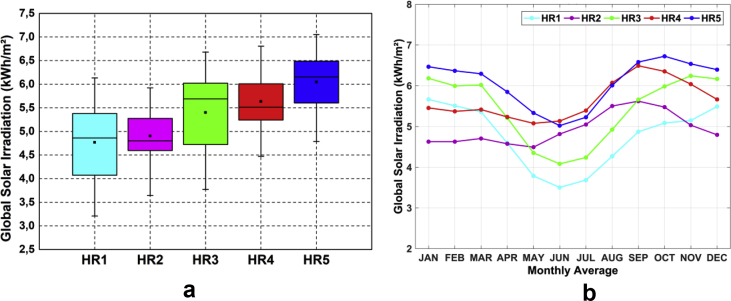
(a) The box plot is presenting the variability of the annual mean of global solar irradiation at the surface in the five clustered areas of NEB exhibiting distinctive seasonal and spatial patterns; (b) Seasonal variability of the monthly average of global solar irradiation in the same areas of NEB.

Fig. 4a presents the box plot of the annual average of the surface solar irradiation in all five areas. The dataset demonstrates that the incoming solar energy is higher in HR5 than all the other areas of NEB. In contrast, the inter-annual variability of solar irradiation in HR2 is the lowest. Fig. 4b presents the plot for seasonal variability of the monthly average of the surface solar irradiation in all five areas. The monthly average in HR2 is smaller than any other area in NEB. By the other side, the amplitude of the seasonal cycle in HR1 is the highest of the NEB.

The research results and conclusions were published in the article: “The Seasonal Variability and Trends for the Surface Solar Irradiation in the Northeastern Region of Brazil” [1].
